# Prognostic values of red blood cell distribution width, platelet count, and red cell distribution width-to-platelet ratio for severe burn injury

**DOI:** 10.1038/s41598-017-13151-3

**Published:** 2017-10-20

**Authors:** Le Qiu, Chen Chen, Shi-Ji Li, Chao Wang, Feng Guo, April Peszel, Sheng Liu, Fei Wang, Ye-Xiang Sun, Yong-Jie Wang, Xu-Lin Chen

**Affiliations:** 1 Department of Burns, the First Affiliated Hospital of Anhui Medical University, Hefei, Anhui 230022 China; 2 Department of Burns, Rui Jin Hospital, Shanghai Jiao Tong University, Shanghai, China

## Abstract

Red blood cell distribution width (RDW), platelet count (PLT), and a RDW-to-PLT ratio (RPR) have been associated with inflammatory activity and adverse outcomes in many diseases. This study has aimed to investigate the association between these indicators and the mortality rate of severe burn patients. From 2008 to 2014, 610 cases of severe burn patients from two burn centers in eastern China were enrolled in this study. Eighty-eight patients died within 90 days after admission. The RDW, PLT, and RPR were studied through Cox regression analysis on the 3rd and 7th day. The RDW, PLT, and RPR values on the 3rd and 7th day were significantly associated with the outcomes of severe burn patients (P < 0.01). High RPR was significantly associated with a 90-day mortality rate at the two time points. However, the RDW and PLT did not provide independent predictive values. Our results indicated that the RPR values on the 3rd and 7th day were associated with the mortality rates of severe burn patients (P < 0.01). Meanwhile, the RDW and PLT values at these time points failed to provide independent values for burn mortality prediction. Thus, the RPR can serve as an independent and novel marker for mortality rates prediction in severe burn patients.

## Introduction

A severe burn is a common and aggressive acute traumatic injury^1^. Due to the advancements of fluid resuscitation, early surgical intervention, nutritional support, and valid infection control, the mortality and morbidity rates of severe burn patients have declined^2^. However, a severe burn patient with advanced age, large total body surface area (%TBSA), and inhalation injury remain to have increased the risk of substantial complications and death^3,4^. Burn cases feature emergency, varied causes, and significant individual differences. Frequently, patients with same TBSA and depth of burn have different outcomes^5^. Given that the general physical response to burns is diverse, we attempted to identify a reliable parameter to trace the general clinical course, particularly the pathological course of inflammation. However, existing injury scores, such as burn injury severity score and Ryan Score, have failed to demonstrate the severity of inflammation^6^. Moreover, inflammatory markers, such as C-reactive protein and procalcitonin, during the early postburn stage are not correlated with the outcome of severe burn injury^4,6,7^.

RDW and PLT are components of the complete blood count (CBC), which is one of the most extensively applied noninvasive laboratory tests. RDW has been used to diagnose and classify anemia by analyzing the size of erythrocytes. An increasing number of studies have evaluated the association between RDW and mortality rates or other complications in various disease states, such heart failure, critical illness, trauma, and sepsis^7–11^. Meanwhile, PLT typically decreases considerably in the first week of a severe burn injury, making thrombocytopenia a prognostic factor for patients with severe burns^6^. Conjunctively the RDW-to-PLT ratio (RPR) is a simple index used to predict significant fibrosis and cirrhosis in chronic hepatitis B patients^8^. Other studies have indicated that RPR is a valuable prognostic marker of inflammation in acute pancreatitis and myocardial infarction with acute ST segment elevation^12,13^.

Few studies illustrated the correlation of RDW, PLT, and RPR with severe burn injury. We herein first investigated the prognostic values of RDW, PLT, and RPR in severe burn patients to provide a simple parameter for their outcome prediction.

## Results

### Patient demographics

We included 652 patients in the study, among which 610 had severe burns. A total of 88 patients died within the 90 days after the initial injury, and 33 patients died in the hospital on the 3rd and 7th day postburn.

Seventeen variables were regarded as potential predictors of outcomes (Table 1). The data were then classified into day 3 and day 7 datasets to reflect the association of the laboratory variables at different time points with the endpoint.

**Table 1:** Demographics and clinical characteristics of patients from follow-up results on days 3 and 7.

	Day 3		P	Day 7		P
	Survivor	Non-survivor		Survivor	Non-survivor	
Demographics						
Number of patients	522	88		522	55	
Age (years)	43.58 ± 15.11	53.52 ± 18.42	0.000	43.58 ± 15.11	52.55 ± 17.98	0.000
Gender (M/F)	384/138	64/24	0.087	384/138	41/14	0.875
Clinical variables						
BI	33.46 ± 17.30	49.61 ± 25.12	0.000	33.46 ± 17.30	52.58 ± 25.99	0.000
Inhalation injury, n (%)	131(25.1)	53(60.2)	0.000	131(25.1)	34(61.8)	0.000
Mechanical ventilation, n (%)	92(17.6)	51(58.0)	0.000	92(17.6)	37(67.3)	0.000
Surgery during the first week, n (%)	302(57.9)	31(35.2)	0.000	302(57.9)	26(47.3)	0.132
LOS, days, mean ± SD	37.67 ± 26.19	12.84 ± 11.98	0.000	37.67 ± 26.19	17.95 ± 12.63	0.000
Laboratory variables						
WBC (×10^9^)	11.57 ± 5.33	12.97 ± 8.66	0.146	13.77 ± 6.35	13.83 ± 8.85	0.960
Neutrophils, ×10^9^/l	9.58 ± 4.83	11.04 ± 7.73	0.090	11.49 ± 5.86	11.78 ± 7.93	0.735
Lymphocytes, ×10^9^/l	1.10 ± 0.56	1.00 ± 0.53	0.123	1.27 ± 0.82	1.19 ± 0.75	0.448
RBC (×10^12^)	4.30 ± 0.81	4.07 ± 0.88	0.017	3.51 ± 0.64	3.08 ± 0.58	0.000
Hemoglobin (g/L)	130.62 ± 26.25	124.34 ± 27.80	0.040	105.97 ± 20.48	93.56 ± 17.22	0.000
MCV	88.49 ± 5.61	89.16 ± 4.58	0.292	87.96 ± 5.68	89.86 ± 4.84	0.017
MHC	30.34 ± 2.37	30.49 ± 1.71	0.574	30.18 ± 1.67	30.42 ± 1.55	0.312
MCHC	343.03 ± 12.32	338.67 ± 35.22	0.253	342.20 ± 11.09	338.72 ± 14.72	0.094
RDW	13.38 ± 1.08	14.00 ± 1.36	0.000	13.74 ± 1.34	14.37 ± 1.36	0.001
PLT	132.73 ± 70.95	97.07 ± 73.71	0.000	207.42 ± 93.66	137.99 ± 75.96	0.000
RPR	0.134 ± 0.087	0.226 ± 0.190	0.000	0.083 ± 0.045	0.146 ± 0.102	0.000

Note: All the variables measured on the 3^rd^ day and 7^th^ day postburn received Student’s t-test or Pearson’s χ2 test. Data were presented as mean ± SD, or number (%). BI indicates burn index; WBC, white blood cell count; RBC, red blood cell count; MCV, mean corpuscular volume; MHC, mean corpuscular hemoglobin; MCHC, mean corpuscular hemoglobin concentration; RDW, red cell distribution width; PLT, platelet count; RPR, RDW to PLT ratio, and LOS, length of hospital stay. Three patients were lost to the 90-day follow-up.

The following parameters suggested significant differences between survivors and non-survivors on days 3 and 7: age, BI, inhalation injury, mechanical ventilation, red blood cell (RBC) count, hemoglobin, RDW, PLT, and RPR.

Surgery during the 1st week and neutrophils of the survivors were only regarded as significantly different from those of non-survivors on day 3. In the day 7 dataset, the MCVs of survivors and non-survivors were significantly different from each other.

No significant difference was observed between survivors and non-survivors in terms of gender, white blood count, lymphocytes, mean corpuscular hemoglobin, and mean corpuscular hemoglobin concentration on the 3rd and 7th day postburn.

### ROC curves of RDW, PLT, and RPR and adverse outcome in severe burn injury

**Figure 1:**
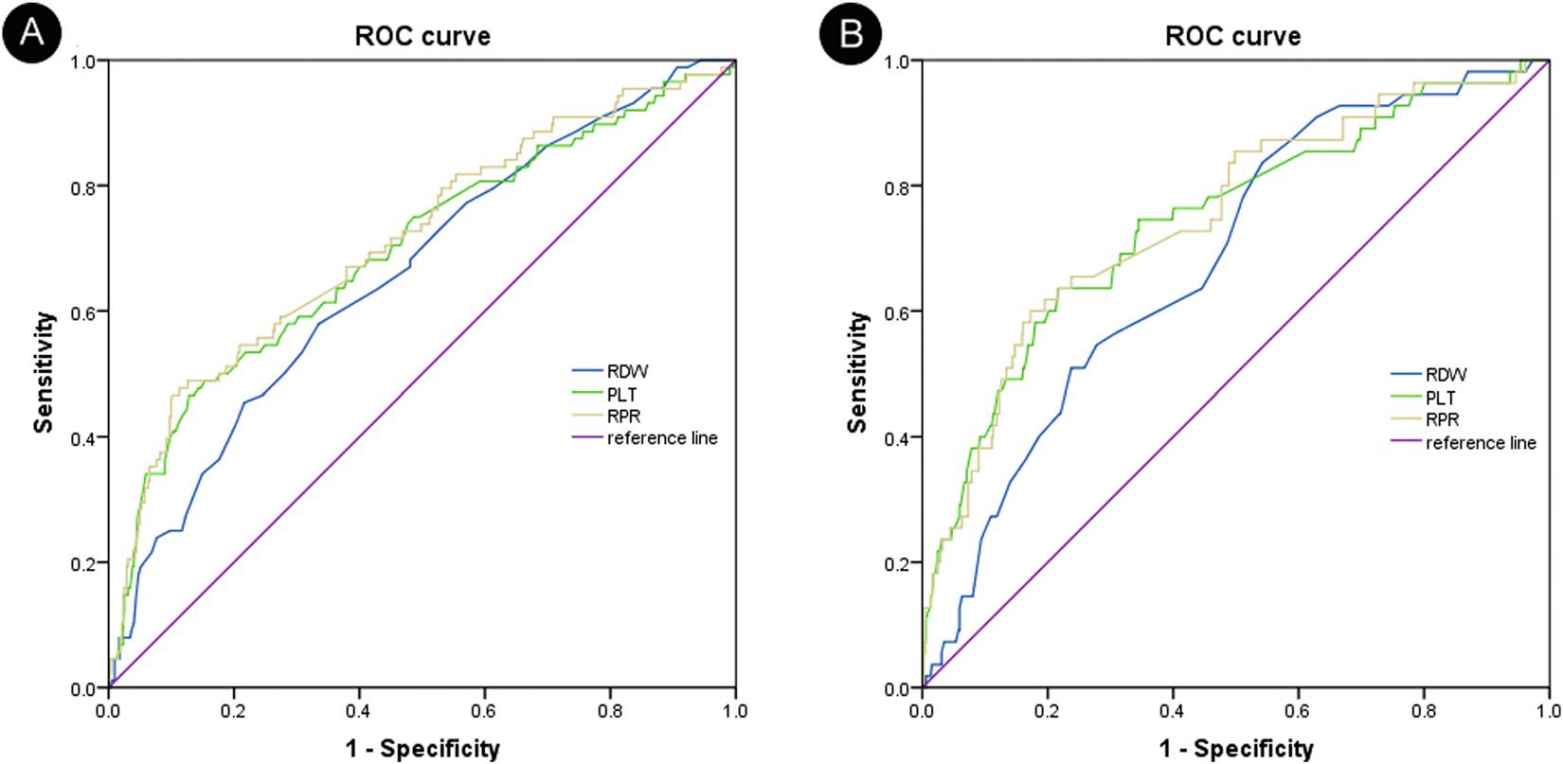
(**A**) Receiver operating characteristic curve of RDW (AUC = 0.659; 95% CI: 0.597–0.720), PLT (AUC = 0.697; 95% CI: 0.632–0.761), and RPR (AUC = 0.712; 95% CI: 0.649–0.774) on day 3 for prediction of adverse endpoint at 90 days. (**B**) Receiver operating characteristic curve of RDW (AUC = 0.681; 95% CI: 0.613–0.750), PLT (AUC = 0.744; 95% CI: 0.670–0.818), and RPR (AUC = 0.750; 95% CI: 0.678–0.821) on day 7 for the prediction of adverse endpoint at 90 days.

The RDW and RPR values were considerably high in patients with adverse outcomes in the day 3 and day 7 datasets, whereas the PLT values were low. As shown in Table 2, the areas under the ROC curves of the RDW, PLT, and RPR for adverse outcome prediction in the day 3 dataset were 0.659 (95% CI: 0.597–0.720), 0.697 (95% CI: 0.632–0.761), and 0.712 (95% CI: 0.649–0.774), respectively, while 0.681 (95% CI: 0.613–0.750), 0.744 (95% CI: 0.670–0.818), and 0.750 (95% CI: 0.678–0.821) in the day 7 dataset. The maximum value of Youden index was used as the criterion for selecting the optimum cut-off point to divide the variables to the high value group and the low value group. The Youden index was represented by the formula Youden index = sensitivity + specificity – 1. RPR ≥ 0.197 (day 3) and 0.108 (day 7) were statistically significant for adverse outcome prediction. In the day 3 dataset, the sensitivity was 47.7% and the specificity was 88.7%; corresponsively 58.9% and 82.7% in the day 7 dataset. For RDW, the cutoff point was 13.5 (day 3) and 13.6 (day 7). The day 3 dataset had an RDW sensitivity of 58.0% and specificity of 66.5%; while the day 7 dataset, 89.3%, and 49.5% respectively. For PLT, the cutoff point was 70.0 with 46.6% sensitivity and 66.2% specificity (day 3) and 143.0 with 63.6% sensitivity and 66.2% and 78.4% specificity (day 7). The prognostic values of RDW, PLT, and RPR assessed through their ROC curves were shown in Fig. 1.

**Table 2:** Diagnostic information for the prediction of mortality on days 3 and 7 according to RDW, PLT, and RPR.

Variable	Optimal cutoff point	Sensitivity (%)	Specificity (%)	AUC (95% CI)
Day 3				
RDW (%)	13.5	58.0	66.5	0.659 (0.597–0.720)
PLT	70.0	46.6	66.2	0.697 (0.632–0.761)
RPR	0.197	47.7	88.7	0.712 (0.649–0.774)
Day 7				
RDW (%)	13.6	83.9	45.9	0.681 (0.613–0.750)
PLT	143	63.6	78.4	0.744 (0.670–0.818)
RPR	0.108	58.9	82.7	0.750 (0.678–0.821)

Note: CI indicates confidence interval. The maximum value of Youden index (sensitivity + specificity −1) was used as the criterion for selecting the optimal cutoff point.

### Survival analysis for predicting severe burn mortality rates

**Figure 2:**
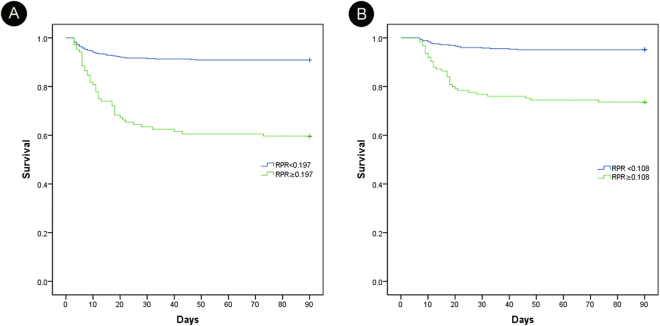
(**A**) Kaplan–Meier survival curves for RPR higher or lower than 0.197 at day 3. (**B**) Kaplan–Meier survival curves for RPR higher or lower than 0.108 on day 7.

The RDW, PLT, and RPR of days 3 and 7 are significantly correlated with the adverse outcomes of severe burn patients according to the univariate regression analysis results (P < 0.01; Table 3). However, the RDW and PLT did not provide independent predictive values in the multivariate Cox regression, while the RPR ≥ cutoff value was significantly associated with 90-day mortality rate (day 3 cutoff point = 0.197, HR = 2.385, 95% CI: 1.006–1.104, P = 0.001; day 7 cutoff point = 0.108, HR = 2.370, 95% CI: 1.320–4.254, P = 0.004). In the multivariate Cox regression analysis, the advanced age, high BI, inhalation injury (day 3), mechanical ventilation, surgery during the 1st week, neutrophils (day 3), and RBC count (day 7) were entered as independent variables (P < 0.01) (Fig. 2).

**Table 3:** Results of univariate and multivariate Cox proportional-hazards regression for the analysis of the effects of baseline variables on an adverse endpoint.

Variable	Day 3				Day 7			
	Univariate		Multivariate		Univariate		Multivariate	
	HR (95%)	P	HR (95%)	P	HR (95%)	P	HR (95%)	P
Advanced age^a^	3.973 (2.514–6.279)	0.000	3.251 (1.986–5.332)	0.000	4.001 (2.199–7.279)	0.000	3.511 (1.845–6.684)	0.000
High BI^b^	3.258 (2.138–4.966)	0.000	2.070 (1.215–3.529)	0.007	4.343 (2.544–7.417)	0.000	2.475 (1.340–4.571)	0.004
Inhalation injury	3.533 (2.303–5.419)	0.000	1.999 (1.218–3.280)	0.006	3.757 (2.177–6.484)	0.000		NS
Mechanical ventilation	4.468 (2.921–6.835)	0.000	1.966 (1.108–3.488)	0.021	6.610 (3.753–11.645)	0.000	3.401 (1.761–6.570)	0.000
Operation during the first week^c^	3.029(1.947–4.711)	0.000	3.128 (1.963–4.985)	0.000	2.116 (1.241–3.609)	0.006	3.002 (1.678–5.371)	0.000
Neutrophils	1.038 (1.006–1.071)	0.021	1.043 (1.013–1.075)	0.005		NS		
RBC	0.741 (0.579–0.949)	0.017		NS	0.427 (0.282–0.646)	0.000	0.576 (0.354–0.938)	0.026
Hemoglobin	0.992 (0.985–1.000)	0.043		NS	0.977 (0.965–0.990)	0.000		NS
MCV		NS			1.105 (1.036–1.179)	0.002		NS
RDW (≥cutoff value)	2.398 (1.569–3.663)	0.000		NS	3.879 (1.896–7.973)	0.000		NS
PLT (≤cutoff value)	4.025 (2.645–6.124)	0.000		NS	4.591 (2.644–7.971)	0.000		NS
RPR (≥cutoff value)	4.563 (3.000–6.940)	0.000	2.385(1.433–3.968)	0.001	5.094 (2.962–8.759)	0.000	2.370 (1.320–4.254)	0.004

Note: Statistic significant factors in Table 1 were analyzed in univariate and multivariate regression analysis, respectively. Advanced age was defined as age ≥65 years. High BI was defined as burn index ≥50. The cutoff values for RDW, PLT, and RPR were shown in Table 2. NS: Non-significant.

Divided by the optimal RPR cutoff values, the Kaplan–Meier plots for the two categories at two time points indicated that RPR values of equal to or more than the cutoff value have lower survival rates than the RPR values less than the cutoff value (Fig. 2). On day 3, the 90-day mortality rate of the low RPR group was 8.1% and that of the high RPR group was 40.4%. On day 7, the mortality was 4.9% in the low RPR group and 26.4% in the high RPR group. The difference between the low and high RPR groups in terms of the difference in time points was statistically significant (P < 0.01).

## Discussion

The release of inflammatory mediators after a burn injury induces massive capillary losses and following high-volume fluid resuscitation often results in coagulopathy. Inflammation and coagulation play an important role in the pathogenesis of microvascular disorders after severe burns and increase the risk of sepsis or death^14,15^. Routinely conducted during admission, CBC analyses contain several interesting parameters that can be used and can be done without any without additional cost. Of these parameters, RDW and PLT have been observed as independent risk factors for various groups. RDW represents the heterogeneity of peripheral circulating red blood cells by measuring erythrocyte size variability and thus it is often used as an indicator to differentiate anemia. Several studies suggested the importance of RDW as a prognostic value in general populations^9^ and numerous medical conditions, such as cardiovascular diseases^10–12^, infectious^7,16,17^, and inflammatory diseases^8,13,18^. Accumulated evidence showed the association between RDW and the following factors: inflammation, oxidative stress, malnutrition, deterioration of renal function, and anemia of different etiologies. Ghaffari^19^ evaluated the association between the accelerated aging of hematopoietic stem cells and several oxidative stress molecules, such as reactive oxygen species, super-oxide dismutase, and glutathione peroxides. They revealed that increasing RDW values indicate these abnormalities. Thus, RDW has been reported as a prognostic marker for the general health condition, particularly in patients with chronic diseases or those who are critically ill^20,21^. Peng and his colleagues^22^ also believed that increased RDW was related to suboptimal health status, which involves impairment of red cell generation. However, the exact mechanism underlying this association remains unclear.

Unfortunately, RDW fails to infer prognostic information in the absence of other specific inflammation indicators. As it was shown in Table 1, though the RDW values of non-survivors were higher than those of survivors, the existing data showed no predictive value between RDW and adverse outcomes of severe burn patients.

Meanwhile, the effect of platelets in the early stage of severe burn injury has been investigated for many years. The application of platelet-rich plasma on severe burn treatment was based on the theory that activated platelets would release growth factors, such as fibroblast and epidermal growth factors, which contributed to wound healing in multiple ways^23^. Through clinical monitoring, Guo Feng *et al*.^6^ found that the PLTs of severe burn patients decrease considerably in 1–2 weeks postinjury. Distinctly low PLTs also strongly predicted the poor outcome of operations^24^. In the present study, a substantial decline in PLT was observed on the 3rd day postburn, and the PLT change during the first 10 days was lower in non-survivors than in survivors.

The CBC parameters in the 1st day after severe burn injury may be interfered by shock, acute stress reaction, and advanced treatments. The RDW and PLT at different time points were diverse in terms of hemoconcentration and erythrocyte damage after burn injury.

RDW and PLT are good predictors for severe burn according to the univariate analysis results (Table 3). However, neither the association between RDW and inflammation nor the correlation between PLT and coagulation can entirely explain the mortality and morbidity rates in severe burn injuries. Considering both parameters in the analysis can reflect the generalized health information after severe burn injury, and the change among different time points may reflect the effect of the current therapeutic regimen.

RPR has been regarded as an important indicator of hepatic fibrosis and cirrhosis prediction in chronic hepatitis B patients^8^. Recently, it has been considered as a novel index marker that reflects inflammation severity, by combining the prognostic advantages of RDW and PLT.

A previous study reported that increased RDW at days 1, 4, and 8 were associated with the severity of and mortality from sepsis^7^. Given that severe burn patients regularly received massive fluid resuscitation in the first 48 hours, we investigated the correlation on days 3 and 7. So far, no evidence exerted the value of RDW during days 3 and 7 as a predictor of adverse outcome in burn patients. We also investigated the prognostic value of RDW during the time of admission and found no positive results (data not shown). As a novel biomarker, a high RPR level on days 3 and 7 represented an adverse correlation with severe burn injury. Meanwhile, the RPR level at admission was found to have no relevance to the outcome (data not shown).

As far as we know, this is the first paper that simultaneously explores the association between the mortality rate and RDW, PLT, as well as RPR in two significant time points post-burn. This retrospective study was performed in two regional specialized burn centers in China, and included a relatively large number of samples. To reduce possible interference with the results, we adjusted the known risk factors into the univariate and multivariate regression analyses. The risk factors were age, % TBSA, inhalation injury, mechanical ventilation, early surgery, and related CBC components, which were crucial to the mortality rates of severe burn injury. Non-surviving patients had a higher RDW and RPR and lower PLT compared to the survivors after burn injury that covered ≥30% TBSA. The RDW and PLT resulted in no prognostic values in severe burn injury, but the RPR on days 3 and 7 turned out to be associated with the endpoint. According to our results, RPR was an independent prognostic marker for the 90-day mortality rate during the two time points, whereas the RDW and PLT were not. We surmised that these findings offered significant clinical applications.

Nonetheless, several limitations hindered our study. First, the retrospective nature of the design limited the research. Despite the large number of patients (610) included in the study, the participants may not be representatives of the general population, because the patients enrolled in the study came from eastern China only. Second, this study failed to include other risk factors, such as race, ethnicity, economic status, and infection. Third, the continuous fluctuations in RDW, PLT, and RPR values were not investigated. These fluctuations may be necessary for the formulation of a comprehensive conclusion.

## Conclusion

Aggressive fluid resuscitation and other medical interventions have alleviated the effects of shock and acute stress reactions during the 48-hour postburn period. Our results indicated that RPR can be used as a simple and novel independent biological marker to predict mortality rates in burn injuries. Meanwhile, although RDW and PLT were associated with the risk of death according to the univariate analyses, these parameters had no value on the endpoint of severe burns during the early stage. Thus, they failed to serve as independent prognostic markers for severe burn injuries. The early measurement of RPR during days 3 to 7 after burn injury is quite important for burn severity evaluation.

## Methods

### Patients

Between January 2008 and December 2015, the retrospective study was performed on 652 severe burn patients admitted to the First Affiliated Hospital of Anhui Medical University and Rui Jin Hospital of Shanghai Jiao Tong University. All severe burn patients had been routinely informed that their demographics, clinical and laboratory data may be used for research and teaching purpose. Informed consent forms were used containing benefits and risks of participating, availability of counseling services, voluntary participation. All procedures were approved by the Human Subjects Review Board of Anhui Medical University Hospital, Hefei, Anhui. All methods were carried out in accordance with relevant guidelines and regulations.

The inclusion criteria were as follows: (1) age of 18 years or older, (2) %TBSA burned of ≥30%, (3) initial fluid resuscitation with the China formula within 8 h after the burn injury, and (4) more than 3 days of hospital stay. The excluded patients had known pre-existing cardiac disease, kidney disease, or other conditions known to alter RDW or PLT, such as hemolytic anemia, bone marrow arrest, or other inflammatory diseases. Three patients who were lost to the 90-day follow-up after injury were also excluded.

The China formula is as follows: 1.5 ml/kg/%TBSA + 5% dextrose solution 2000 ml, and 1.5 ml/kg/%TBSA = lactate Ringer’s solution 1.0 ml/kg/%TBSA + fresh-frozen plasma 0.5 ml/kg/%TBSA^25^. The formula was modified according to the physiological response of the patient. Half of this volume was administered in the first 8 h, and the remaining half was administered during the next 16 h. The burn index (BI) had the formula BI = %TBSA of third-degree burns + ½ %TBSA of second-degree burns.

### Study design

We performed this retrospective study on patients admitted to the burns departments of the First Affiliated Hospital of Anhui Medical University and Rui Jin Hospital of Shanghai Jiao Tong University. Medical notes were used to retrospectively record the patients. Their blood samples were routinely collected for CBC between 5 am and 7 am during the 3rd and 7th day postburn. The demographic data and outcomes of the patients were downloaded from the medical and follow-up records. The medical records included demographics, clinical variables, and surgical records during the 1st week. The clinical variables included the BI, presence of inhalation, and mechanical ventilation. The medical or follow-up records were used to document the time from injury to adverse events. The follow-up-to-evaluate endpoint was defined as the time interval from admission to death or discharge.

### Statistical analysis

The patients were grouped according to the endpoint and were presented at two time points. Data were presented as the mean ± standard deviation (SD) or frequency (%). Intergroup comparisons were performed with Student’s t-test or Pearson’s χ2 test. The prognostic values of RDW, PLT, and RPR were determined through receiving operating characteristic (ROC) curves. Every variable was distributed to the high value group and the low value group. The boundary was determined by a threshold to show statistically significant differences between two groups in outcome prediction with optimal sensitivity and specificity. The maximum value of Youden index was used as the criterion for selecting the optimum cut-off point. RDW and RPR ≥ cutoff points, as well as PLT ≤ cutoff points, were significantly associated with the adverse outcome prediction.

The independent effects of the variables on the adverse events at different time points were calculated using the Cox multivariate proportional hazard regression. The hazard ratios and 95% confidence intervals (95% CI) were calculated as measures of the clinical impacts of the markers. The survival curves of the parameters were designed through the Kaplan–Meier method and according to the results of the Cox regression. A P value of < 0.05 was considered statistically significant. Statistical analyses were performed in SPSS 22.0 (SPSS Inc., Chicago, IL, USA).
